# Recent advances in understanding the genetic resources of sheep breeds locally-adapted to the UK uplands: opportunities they offer for sustainable productivity

**DOI:** 10.3389/fgene.2015.00024

**Published:** 2015-02-12

**Authors:** Dianna Bowles

**Affiliations:** ^1^Department of Biology, University of YorkYork, UK; ^2^The Sheep Trust, University of YorkYork, UK

**Keywords:** biodiversity, adaptive fitness, sheep, genomics, farm animal genetic resources

## Abstract

Locally adapted breeds of livestock are of considerable interest since they represent potential reservoirs of adaptive fitness traits that may contribute to the future of sustainable productivity in a changing climate. Recent research, involving three hill sheep breeds geographically concentrated in the northern uplands of the UK has revealed the extent of their genetic diversity from one another and from other breeds. Results from the use of SNPs, microsatellites, and retrovirus insertions are reviewed in the context of related studies on sheep breeds world-wide to highlight opportunities offered by the genetic resources of locally adapted hill breeds. One opportunity concerns reduced susceptibility to Maedi Visna, a lentivirus with massive impacts on sheep health and productivity globally. In contrast to many mainstream breeds used in farming, each of the hill breeds analyzed are likely to be far less susceptible to the disease threat. A different opportunity, relating specifically to the Herdwick breed, is the extent to which the genome of the breed has retained primitive features, no longer present in other mainland breeds of sheep in the UK and offering a new route for discovering unique genetic traits of use to agriculture.

## Introduction and context

The importance of locally adapted livestock breeds is becoming recognized through their contribution to food security in marginal land areas of the world. Reviews by the FAO and other international agencies highlight the crucial significance of these breeds, both as sustainable resources of food but also as living reservoirs of biodiversity, providing genetic adaptive fitness traits to improve mainstream agriculture (FAO, 2009; Hoffmann, 2013).

In the UK, the locally adapted hill breeds of sheep are typically farmed on Disadvantaged or Severely Disadvantaged Areas for agricultural production (Defra, 2012). These breeds are characterized by their ability to thrive in the harsh environments of mountains, fells, and moorlands, rearing lambs on low inputs of feed and management. The breeds are not rare and exist in many tens of thousands. But, due to their adaptation to local environments, each breed is concentrated within a single region of the UK (Carson et al., 2009). In these regions, the sheep continue to be commercially farmed and contribute to the heritage and economies of their guardian communities.

The extent of the breed's geographical concentration, leads to their susceptibility and disproportionate losses if an infectious disease enters their region. This issue was first recognized in the UK during the Foot and Mouth Disease (FMD) epidemic of 2001 which started in the northwest of England and disproportionately affected livestock farmed in that region [Archive.defra.gov.uk/foodfarm/farmanimal/diseases/atoz/fmd/2001; www.royalsoc.ac.uk (2004). Policy document 25/04 ISBN 0854036105 Royal Society Infectious Disease in Livestock Inquiry: Follow Up review]. Losses to the Herdwick sheep breed were of particular concern, which led to the setting up of the first national gene bank for sheep and a government commitment to survey the precise level of geographical concentration that existed amongst commercially farmed regional breeds.

The Sheep Trust, a national UK charity, working with Sheep Breeder Associations (SBAs) and more than 1000 breeders, geo-referenced individual flocks of 12 regional breeds (Carson et al., 2009). The data showed certain of these Heritage Breeds were indeed extremely concentrated with for example, up to 95% of the Herdwicks numbering some 47,000 animals, tightly clustered within 23 km of the breed's mean center in the Lake District National Park of northern England.

Endangerment of the genetic resources of livestock may arise through disease but significantly, for the hill breeds of sheep throughout Europe, changes in the policies of national governments and EU regulations are also leading to new risks (www.publications.parliament.uk/pa/cm201011/cmselect/cmenvfru/556/556.pdf; http://publications.naturalengland.org.uk/publication/410700). Historically, sheep numbers increased as a reflection of headage payments in the EC's Common Agricultural Policy (CAP) and this undoubtedly led to some overgrazing of the uplands. Changes to CAP then switched to subsidy payments based on land area rather than numbers of animals farmed. This is leading to major reductions in hill sheep numbers, since farmers are no longer being paid on flock sizes. In some areas, agri-environment schemes to promote rejuvenation of the vegetation are also decreasing sheep numbers further with zero stocking of the hills for certain times of the year.

Recognition of risk to a sheep breed's genetic resources through geographical concentration has recently been accepted in policies for breed protection and conservation (www.gov.uk/government/uploads/system/uploads/attachment_data/file/254127/uk-breeds-at-risk.pdf). However, it is also crucial to determine the genetic distinctiveness of each breed. This is an indicator of the biodiversity deserving protection and is also a potential route to identifying adaptive fitness traits that can underpin the future sustainability and security of food production.

This mini review describes new insights gained from a recent genetic study of Herdwicks, Rough Fell and Dalesbred, three hill breeds of the UK (Bowles et al., 2014). These locally adapted breeds, each geographically concentrated in the northern uplands of England have not been previously analyzed. The new findings add to our current understanding of sheep genetic resources world-wide.

## Analytical tools

Molecular genetic studies of sheep breeds have used a number of approaches to analyze breed origins, relatedness, distinctiveness and selection (reviewed in Groeneveld et al., 2010; Lenstra et al., 2012).

These include single nucleotide polymorphisms (SNPs) using a candidate gene approach, as well as the increasingly widespread application of the *OvineSNP50BeadChip* that is leading to considerable advances (for example, Pariset et al., 2006a,b; Kijas et al., 2009, 2012; Johnston et al., 2011, 2013; Heaton et al., 2012, 2013, 2014; Riggio et al., 2013; Zhang et al., 2013; Fariello et al., 2014; Gutiérrez-Gil et al., 2014; Hazard et al., 2014; Lv et al., 2014; McRae et al., 2014; Periasamy et al., 2014), including those addressing global sheep diversity undertaken by the International Sheep Genomics Consortium (Kijas et al., 2009, 2012). These studies complement and extend the earlier use of microsatellites to investigate variation and population structure (for example, Alvarez et al., 2005; Tapio et al., 2005a,b, 2007; Lawson Handley et al., 2007; Peter et al., 2007), particularly in the study of European breeds. Also, an unrelated approach based on retrotyping, has enabled the origins and routes of domestication to be investigated through analyzing insertions of a panel of endogenous Jaagsiekte sheep retroviruses (enJSRVs) into defined sites of the genome (Arnaud et al., 2007; Chessa et al., 2009).

A selection of these approaches was applied to study the genetic resources of the three geographically concentrated hill breeds, each farmed in adjacent regions of the UK northern uplands. (Bowles et al., 2014; http://www.herdwick-sheep.com; www.dalesbredsheep.co.uk; www.roughfellsheep.co.uk). Equal numbers of registered rams from each of the breeds comprised the populations selected because provenance, familial origins and lack of relatedness could be confirmed. Also, it was reasoned that rams contribute the major genetic resource to a breed, whilst forming only a small fraction of the total breed numbers.

## Insights gained

### Reduced susceptibility to maedi visna

Small ruminant lentiviruses infect millions of animals globally, with Maedi-Visna (MV) a major disease, reducing sheep health and productivity as well as lifespan. Studies by Heaton et al. (2012, 2013) have clearly demonstrated that variations in the gene encoding TMEM154, an ovine transmembrane protein, are associated with infection of sheep to the lentivirus. Polypeptide variants with the presence of glutamate (E) at position 35 in the ancestral, full length version of the protein are associated with increased susceptibility to the lentivirus, whereas lysine (K) at position 35, or deletion mutants are associated with reduced susceptibility (Heaton et al., 2012).

Heaton reported the average frequency of the highly susceptible *TMEM154* alleles was 0.51 across 74 sheep breeds world-wide (Heaton et al., 2013). Significantly, >25% of those analyzed, including some mainstream breeds such as the Scottish Texel, showed a frequency of above 0.8 indicating a major latent health and welfare risk for the global sheep industry. This contrasts with the recent data on the three hill breeds (Bowles et al., 2014), with frequencies for the susceptible allele ranging from 0.26 to 0.42, suggesting a lower than average risk of MV infection and a much greater likely resilience to the disease threat.

In the UK, flocks of continental breeds such as Texels (www.texel.co.uk), stringently accredited for the absence of MV, represent a major component of the mainstream sheep industry. Genetic resistance of the hill breeds to lentivirus infections offers an alternative route to achieve an infection-free status for the national flock and an important reason to protect and continue to make use of the genetic resources of these locally adapted breeds.

### Lack of in-breeding amongst locally adapted breeds

Panels of microsatellites have been used with great effect to study many aspects of sheep breeds world-wide (Alvarez et al., 2005; Tapio et al., 2005a,b, 2007; Lawson Handley et al., 2007; Peter et al., 2007). These have included large-scale comparisons such as 57 breeds across Europe and the Middle East by Peter and co-workers of the ECONOGENE Consortium (Peter et al., 2007) and 32 breeds of Northern Europe by Tapio et al. (2005a), as well as studies on breeds farmed in close geographical proximity, such as in regions of the Baltic and in northern Spain (Alvarez et al., 2005; Tapio et al., 2005b, 2007).

In the study of the three UK hill breeds, a subset of microsatellites were applied to investigate the possibility of in-breeding within the populations analyzed (Bowles et al., 2014). This is relevant since within the extensive hill farming systems of the uplands, detailed written pedigrees are not maintained and for some breeds, such as the Herdwicks, only rams are formally registered. This informal management of genetic resources is a risk and differs from monitoring in the commercial breeding of mainstream breeds and in the conservation breeding of numerically scarce rare breeds.

However, when the occurrence of heterozygote deficiency within populations per marker per breed was evaluated, no significant inbreeding was revealed. The average local inbreeding coefficient (*F*_IS_) was weakly positive in all cases, with the lowest value of 0.059 in Herdwick and highest in Rough Fell (0.162). These values are within the range previously found for two other northern UK breeds with substantially greater population sizes (Peter et al., 2007), the Swaledale (0.070) and the Scottish Blackface (0.031), indicating the informal practices supported by Breed Societies are currently sufficient to maintain diversity within the genetic pool of each breed.

### Origins and opportunities of a primitive genome

The use of enJSRVs as genetic markers (Arnaud et al., 2007; Chessa et al., 2009) enabled Chessa and co-workers to explore the historical origins and routes of domestication of sheep breeds across the world. They reasoned that given each endogenous retrovirus in a host genome arises from a single irreversible integration event, populations sharing the retrovirus DNA in the same genomic location must be related phylogenetically. Four common retrovirus insertions enabled them to design a classification scheme of “retrotypes” that was applied to 133 breeds globally. Genomes with none of the common insertions were rare and defined as “primitive” since they preceded any of the integration events. Only 10 breeds analyzed world-wide were found to have an abundance of individuals with this primitive characteristic (Chessa et al., 2009).

In the recent study of the hill breeds, very surprisingly, the Herdwick population contained a high proportion of animals with a “primitive” (R0) genome. In contrast, R0 was absent from Rough Fell and Dalesbred, both of which contained abundant retrotypes more typical of other UK breeds.

The presence of R0 individuals retained in the present day Herdwick population, provides an indicator of origins of domestication as well as farming practices over the centuries. The data suggest Herdwicks originate from a common ancestral founder flock to other “primitive” breeds retaining the R0 retrotype. Within the UK only the North Ronaldsay of the Orkney Islands are known to have R0's and to an extreme proportion. Elsewhere, substantial levels of R0's were found in populations in Sweden, Finland and Iceland (Chessa et al., 2009). The fact that R0 individuals continue in these present day breeds most probably reflects the degree of isolation with which they have been farmed since their first introduction. In turn, this may also reflect the original extent of their “fitness” to those harsh northern, upland environments and this fitness has continued to be maintained over the centuries by farming practices.

A second surprising feature shared both by the Herdwicks and the Rough Fell, was the presence of two extremely rare retrovirus insertions in their populations. These insertions were so rare that they were not included in the global retrotype classification and in fact in that earlier study, the pair was only found in Texel breed populations (Chessa et al., 2009). Present day Texels originate from a number of breeds (www.texel.co.uk), none of which were shown to have the rare retrovirus insertions (Chessa et al., 2009). This suggests therefore that it may be a very distant historical association between the two UK hill breeds and current Texels, such as involving a common origin in the form of the Pin-tail ancestral population of Texel Island in the Wadden Sea. This region, now designated as a World Heritage site, was once above sea level and from archeological remains is known to have been a major trading post for Vikings as they moved outwards to colonize the south and west (Besteman, 2004; Knole, 2010). Intriguingly, local folklore in Cumbria has always linked the arrival of Herdwicks to the early Viking settlers (Brown, 2009). The new genetic data suggest that link extends to the Rough Fell, although the lack of any R0 retrotypes in today's Rough Fell population implies a subsequent greater divergence from the common founder flock, compared to the Herdwicks.

## Conclusions and way forward

Genetic diversity in farm animals is becoming recognized as a highly significant resource. With the rapid progress in DNA sequencing technologies and the availability of SNP chips for many major species, genetic breeding is increasingly being used for improvement. As yet however, many of these practical applications continue to focus on production traits and the mainstream breeds of livestock.

Predicted impacts of climate change, increases in food requirement for the world population and economic costs of farming are all contributing to renewed interest in the genetic resources of those breeds that are locally adapted to their farmed habitats. This interest is of relevance to improving a sustainable form of agricultural productivity in both the developed and developing worlds. Low inputs with good feed conversion, hardiness to harsh environments, enhanced resistance to parasites and pathogens, and low management costs are all characteristics of local breeds when farmed extensively in the habitats to which they are adapted (FAO, 2009; Hoffmann, 2013).

In this context, locally adapted sheep breeds are production systems of our food on land that often cannot support any other forms of agriculture. Their ability benefits current food security and provides opportunities for future farming systems that will be required to maintain productivity in the predicted harsh conditions of climate change. Despite the benefits offered by these breeds, the very real risk of their genetic erosion requires urgent action before these living reservoirs of adaptive fitness traits become lost forever.

The first step is to analyze their genetic distinctiveness to demonstrate to policy makers the biodiversity that the sheep breeds contain. The three UK breeds recently studied have not been analyzed previously, but extend earlier molecular studies of other locally adapted and mainstream breeds. A surprisingly broad genetic range was observed for the hill breeds, even though they were farmed in close proximity within adjacent regions of the northern uplands of England.

Next generation sequencing, the use of the *OvineSNP50BeadChip* and global collaborative networks such as the International Sheep Consortium, will provide many more opportunities for large-scale genetic characterization of sheep breeds. Already there are examples of those technologies used to explore traits relevant to adaptation, such as growth characteristics (Zhang et al., 2013), milk production (Gutiérrez-Gil et al., 2014), and social behavior (Hazard et al., 2014). Attention is also focusing on genetic factors important for resisting gastro-intestinal (GI) parasites (Riggio et al., 2013; McRae et al., 2014; Periasamy et al., 2014). Infections from GI nematodes have major impacts on sheep health, welfare and productivity through to economic costs for management input and use of control agents (Nieuwhof and Bishop, 2005) and the problem is worsening through emerging resistance to the major anthelmintic classes (Beech et al., 2011). Selective breeding, whether for resistance or tolerance (Bishop, 2012) is likely to be a major output from future genomic studies.

However, many more data are required linking the different phenotypic traits for potential use in genetic improvement to geographies and the specific ecologies and parasite challenges to which the livestock are adapted. Recent advances in landscape genomics and the bringing together of large data sets combining phenotypic recording, DNA analyses and environmental parameters are providing a new platform to achieve this holistic understanding (Joost et al., 2007, 2013; Colli et al., 2014). Significantly, use of the *OvineSNP50BeadChip* has recently been applied in a large project studying 32 sheep breeds adapted to a wide spectrum of different regional climates (Lv et al., 2014). Two hundred and thirty SNPs were identified with evidence for selection likely due to climate-mediated pressure and 17 strong candidate genes were highlighted to be under environmental adaptive selection. This study confirms the utility of a genomic approach to understand adaptation of sheep to their environments and if now applied to geographically concentrated breeds, such as the Herdwick with its potential abundance of primitive features, there is real potential to uncover the genetic basis of the breed's unique adaptations.

### Conflict of interest statement

The author declares that the research was conducted in the absence of any commercial or financial relationships that could be construed as a potential conflict of interest.
